# The Potential for microRNA Therapeutics and Clinical Research

**DOI:** 10.3389/fgene.2019.00478

**Published:** 2019-05-16

**Authors:** Johora Hanna, Gazi S. Hossain, Jannet Kocerha

**Affiliations:** ^1^ Nova Clinical Research, LLC, Bradenton, FL, United States; ^2^ Department of Chemistry and Biochemistry, Georgia Southern University, Statesboro, GA, United States

**Keywords:** microRNAs, noncoding RNA, epigenetic, ncRNA, preclinical, clinical trials, clinical research

## Abstract

As FDA-approved small RNA drugs start to enter clinical medicine, ongoing studies for the microRNA (miRNA) class of small RNAs expand its preclinical and clinical research applications. A growing number of reports suggest a significant utility of miRNAs as biomarkers for pathogenic conditions, modulators of drug resistance, and/or as drugs for medical intervention in almost all human health conditions. The pleiotropic nature of this class of nonprotein-coding RNAs makes them particularly attractive drug targets for diseases with a multifactorial origin and no current effective treatments. As candidate miRNAs begin to proceed toward initiation and completion of potential phase 3 and 4 trials in the future, the landscape of both diagnostic and interventional medicine will arguably continue to evolve. In this mini-review, we discuss miRNA drug discovery development and their current status in clinical trials.

## Introduction

Groundbreaking results from the Human Genome Project revealed extensive complexity in our DNA sequence and unleashed new opportunities in biomedical and clinical trial studies (Lander et al., 2001; International Human Genome Sequencing Consortium, 2004). Correspondingly, this nucleotide complexity aligns with the multifactorial etiology of many human diseases. Clinical trials that traditionally target single proteins or nodes in biological pathways often fail to reverse pathogenic phenotypes (Kola and Landis, 2004; Hopkins, 2008; Pammolli et al., 2011), further compelling new therapeutic options. As the completion and publication date of the Human Genome Project nears the 20-year mark, the landscape of biomedicine continues to evolve, and likely will for decades to come as massive amounts of sequence data are computationally analyzed and investigated.

Future clinical trials will likely continue to capitalize on the revolutionary findings from the Human Genome Project that RNA transcripts far outnumber protein-encoding genes. This is a significant paradigm shift from the decades-old “central dogma” that one RNA makes one protein. Further, the classes of these nonprotein-coding RNAs (ncRNAs) have vastly expanded with ongoing research studies and now include transcripts such as microRNAs (miRNAs), natural antisense transcripts, Piwi-interacting RNA (piRNAs), and long ncRNAs (lncRNAs) (Bartel, 2009; Washietl et al., 2012; St Laurent et al., 2015). Arguably, miRNAs are one of the most predominantly represented ncRNA groups in clinical research. A typical miRNA is processed from a long primary RNA sequence to a short mature functional transcript around 22 nucleotides in length. A common characteristic of an miRNA is its ability to pleiotropically target potentially hundreds or even thousands of genes, and some function in an organ- or cell-specific manner (Bartel, 2009). Correspondingly, this opens up the possibility of one miRNA candidate having the capability to regulate entire biological pathways that are pathogenically disrupted in a patient. Indeed, miRNAs are proving to be therapeutically advantageous, particularly in health conditions or diseases that may not be provoked by a single genetic link. Although the pleiotropic nature of miRNAs undeniably makes off-target biological effects a concern, similar nonspecific responses have also occurred with traditional therapeutics that target single protein-coding genes. Collectively, small molecule drugs have successfully been pharmacologically designed to pleiotropically target multiple targets or pathways (Hopkins, 2008; McCall et al., 2017), providing further support to miRNAs as future therapeutics.

## Preclinical Validation of miRNA Therapeutics

### Bioinformatic Analysis

Certain mechanistic functions of candidate miRNAs can be assessed bioinformatically and/or *in vitro* prior to testing in preclinical animal models. A growing number of databases are available to predict miRNA regulatory targets (Bartel, 2009; Washietl et al., 2012). Ideally, multiple independent algorithms are used cooperatively for successful prediction of miRNA-binding sites in protein-coding genes and their associated biological networks. One of the first and most consistently published algorithms, TargetScan, came from the laboratory of David Bartel. TargetScan predicts miRNA gene targets based on seed regions that are critical for binding to mRNA, which includes almost all identified miRNA sequences reported in miRBase to date. Moreover, established bioinformatic platforms such as Kegg and IPA/Ingenuity identify putative biological pathways, and in some cases disease states, targeted by miRNAs. There are also computational programs to calculate the free energy between two select RNA sequences of interest, with a lower free energy (typically around −20 or less) suggestive of more stringent binding (Lekprasert et al., 2011; Riffo-Campos et al., 2016). Websites such as https://tools4mirs.org/ provide links to a majority of the currently available software. Future streamlining of clinical research data repositories with miRNA bioinformatic platforms could further facilitate identification and evaluation of therapeutic candidates.

### *In vitro* and *in vivo* Validation

A variety of cell culture platforms are now available to assess the mechanisms, toxicity, and potential therapeutic efficacy of miRNA candidates *in vitro*. Primary cells, immortalized cell lines, and induced-pluripotent stem (IPS) cells are readily available for epigenetic manipulation of target transcripts. IPS cells allow for modulation and monitoring of biological pathways along distinct stem cell lineages from a skin tissue source (Karagiannis et al., 2019; Tsuji et al., 2019). Indeed, vectors and modified oligonucleotides to either overexpress or silence miRNA function are generated for many of these ncRNAs and accessible to biomedical research laboratories. High-throughput capacities to screen and validate bioinformatic predictions *in vitro* have significantly facilitated and expedited preclinical study of putative therapeutic transcripts. Further, with the extensive collection of animal models available commercially or through academic laboratories, *in vivo* validation is increasingly feasible and rapid. Nonhuman primate models have also been successfully incorporated into preclinical miRNA investigations and supported the initiation of clinical trials, such as for the miR-122 repressor, Miravirsen, in the treatment of Hepatitis C Virus (HCV) (Lanford et al., 2010; Janssen et al., 2013).

## miRNAs as Biomarkers in Clinical Medicine

A growing number of reports have shown that subsets of miRNAs may have clinical relevance as biomarkers. These biomarkers can be used to indicate presence of a pathology and even the stage, progression, or genetic link of pathogenesis (Kocerha et al., 2011; Wang et al., 2011, 2015; Weir et al., 2011; Elfimova et al., 2012; Li et al., 2013; Recchioni et al., 2013; Scott et al., 2015; Biswas, 2018; Hu et al., 2018). In certain situations, one miRNA biomarker may be sufficient to identify a health outcome; however, in other cases, a well-defined panel of miRNAs is necessary for increased diagnostic sensitivity and/or specificity. These investigations have been undertaken in preclinical animal models and in populations of humans. For example, studies in a subset of patients with frontotemporal dementia showed distinctive miRNA expression patterns between patients with or without mutations in the gene for progranulin. Further, these distinctive miRNA profiles in some cases were brain region specific to sites of pathology (Kocerha et al., 2011). In this era of rapid access to cutting-edge genome (DNA), transcriptome (RNA), and proteome (protein) data and technology, the continued merging of biomarkers in patient care will undoubtedly enable earlier diagnosis and expedited medical intervention decisions. It is possible that a subset of miRNA biomarkers may also function as viable drug candidates.

To date, a number of registered clinical studies in the clinicaltrials.gov database for miRNA biomarkers have been completed; this includes phase 4 trials that monitored select miRNAs as biomarkers for disease progression in patients receiving FDA-approved drugs. Trials have assessed or are actively recruiting patients to examine the profiles of these ncRNA transcripts in a range of health conditions such as diabetes, coronary heart disease, breast cancer, lupus, epilepsy, depressive disorder, stroke, Addison’s disease, influenza, liver disease, and even toxic exposure to agents such as acetaminophen. Additionally, as miRNAs are ubiquitously expressed throughout the body, they can readily be measured from peripheral blood, tissue biopsies, saliva, urine, cerebrospinal fluid (CSF), and other biological samples (Weber et al., 2010; Gallo et al., 2012; Sun et al., 2014). Notably, as miRNAs are able to cross the blood-brain barrier, they can potentially be quantitated in routine blood, serum, or plasma tests as measures for various neurodegenerative and neurodevelopmental impairments. Indeed, these miRNA diagnostic blood tests to assess brain health are a primary focus of the biotech company DiamiR.

The source of the miRNA candidate influences how accessible it is as a biomarker, how the miRNA is extracted from the biological specimen, and even how it is analyzed. In fluid specimens such as blood, the miRNA content can originate through a few pathways, including extracellular vesicles (exosomes) (Kanlikilicer et al., 2016; Bayraktar et al., 2017; Rashed et al., 2017; Paskeviciute and Petrikaite, 2019). Ongoing investigations continue to probe whether enrichment of exosomes from biological samples or more crude cell-free preparations are more reliable in biomarker measurements; and some reports suggest it may depend on the specific human disease. Vesicle purification can lower the RNA yield and integrity. Correspondingly, because of lower RNA concentration, this can lead to more interindividual variability. Further, a disease-specific alteration in exosome release and clearance, or administration of therapeutics that alter blood volume, can also skew RNA yield (Buschmann et al., 2018). Thus, taking into account disease state and medical treatments may improve biomarker analyses. In addition to cell secretory mechanisms, circulatory RNAs are also derived from cell death, tumor necrosis, or even lipoprotein-mediated transport (Kawaguchi et al., 2016). Levels of miRNAs from biological fluids are routinely quantitated in academic, industry, and hospital laboratory settings by Next-Generation Sequencing, real-time PCR, or microarray platforms and data subsequently normalized to appropriate small RNA controls.

## Clinical Research Studies of miRNAs as Medical Intervention Drugs

This is an exciting time for therapeutic small RNA (less than 200 nucleotides in length) drugs, as the first small-interfering RNA (siRNA) was recently granted FDA approval in 2018. The siRNA drug, Patisiran, is approved for a rare polyneuropathy caused by hereditary transthyretin-mediated (hATTR) amyloidosis and works by binding and degrading the messenger RNA transcript for transthyretin (Kristen et al., 2018; Yang, 2019). Although the emergence of miRNA therapeutics has not yet translated into FDA-approved candidates for medical intervention, candidate drugs are in clinical development or in phase 1 and phase 2 clinical trials. Academic laboratories, biotech companies, and the pharmaceutical industry are all involved in the clinical research efforts. There are biotech companies focused exclusively on advancing miRNA-related drug pipelines, such as Miragen, MiRNA Therapeutics (now Synlogic), and Regulus Therapeutics.

To date, there is reported clinical utility in peripheral tissues for miRNA mimics (to overexpress the transcript) as well as miRNA repressors (to silence the transcript function). Systemic delivery methods of miRNA drugs parallel those of traditional therapeutics with injection or intravenous administration. For cancer-related pathologies, intratumoral injections of miRNA drugs directly into the pathogenic site can enhance target specificity, efficacy, and minimize side effects (Mercatelli et al., 2008; Chen et al., 2015). Arguably, one big hurdle is delivery of drugs to the brain, which has been an ongoing area of investigation for decades even prior to the Human Genome Project publication. Modified micelle, liposome, nanoparticle, intranasal, and other delivery methods have all been tested for blood-brain barrier crossing and delivery with varying levels of success (Garg et al., 2015; Dong, 2018). Although cerebrospinal fluid administration is an option, it has its own efficacy issues and is an invasive procedure with significant risks. Complicating design for delivery of drugs to the brain is that only lipid-soluble small molecules less than 400 daltons are able to cross the blood-brain barrier, macromolecules cannot successfully penetrate.

Putative miRNA drugs have exhibited significant efficacy in a range of health conditions, including cancer, hepatitis C, heart abnormalities, kidney disease, pathologic fibrosis, and even keloid formation. In line with other clinical trials, some miRNA candidates have shown potential, while some have failed and the study was terminated. For example, in 2016, the biotech company MiRNA Therapeutics (now Synlogic) halted phase 1 trials of its miRNA-34 drug mimic, MRX34, for use in cancer after severe adverse events (SAE) were reported in five patients experiencing serious immune responses. Due to the development of SAE, future phase 2 trials of MRX34 for melanoma were also terminated.

Although no miRNA drug candidates have been entered into clinicaltrials.gov database for phase 3 trials to date, there are active earlier phase trials to investigate new miRNA drug candidates in addition to the previously mentioned miR-122/Miravirsen studies. There is ongoing recruitment of patients for a phase 1 trial of the drug MRG 110, a locked nucleic acid (LNA)-modified antisense oligonucleotide to inhibit the function of miR-92. The company miRagen recently announced a second phase 1 trial with MRG 110 with a future potential clinical application toward wound healing as well as heart failure. Miragen also has an active phase 1 study for miR-29 (MRG-201) to treat keloid and scar tissue formation as well as a phase 2 trial for miR-155 (Cobomarsen; MRG-106) for patients with a form of T-cell lymphoma. This year (2019), Regulus announced their new miRNA drug candidate nominee RGLS5579 that targets miR-10b for potential trials in patients diagnosed with glioblastoma multiforme, one of the most aggressive forms of brain cancer with a median survival of approximately 14.6 months (Ghosh et al., 2018). After recent restructuring of Regulus, Sanofi will assume costs for phase 2 trials of Regulus drug candidate RG-012 to silence miR-21 function in patients with Alport syndrome.

The first recently completed phase 1 trial engaging a newer technology termed a “TargomiR” exhibited encouraging results in patients with recurrent malignant pleural mesothelioma or non-small cell lung cancer. In brief, TargomiR delivery vehicles contain an miRNA mimic, bacterially derived minicells, and a targeting moiety (i.e., a specific antibody that recognizes a protein on target cells). In the first human trial of a TargomiR drug, MesomiR-1, the miRNA mimic was the reported tumor suppressing transcript miR-16 and the targeting moiety was an antibody to the epidermal growth factor receptor (EGFR) that is consistently deregulated in lung cancer cells (Reid et al., 2016; van Zandwijk et al., 2017). These studies provide new hope to mesothelioma patients in which less than 10% survive more than 5 years (de Gooijer et al., 2018). Overall, these studies and reports suggest a viable future for miRNA drugs in diseases with no current effective treatments (Table 1).

**Table 1:** Interventional clinical trials for miRNAs.

miRNA gene; drug name	Clinical trial number; phase status	Disease/disorder investigated
miR-34; MRX34	NCT01829971; phase 1 (terminated)	Liver cancer, lymphoma
	NCT02862145; phase 1 (withdrawn)	melanoma
miR-92; MRG 110	NCT03603431; phase 1 (recruiting)	wound healing, heart failure
miR-16; MesomiR-1	NCT02369198; phase 1 (completed)	Mesothelioma, lung cancer
miR-122; Miravirsen	NCT02508090; phase 2 (completed)	Hepatitis C virus
	NCT02452814; phase 2 (completed)	
	NCT01200420; phase 2 (completed)	
	NCT01872936; phase 2 (unknown status)	
	NCT01727934; phase 2 (unknown status)	
	NCT01646489; phase 1 (completed)	
miR-29; MRG-201	NCT03601052; phase 1 (recruiting)	Keloid, fibrous scar tissue formation
miR-21; RG-012	NCT02855268; phase 2 (suspended, sponsor decision)	Alport syndrome
	NCT03373786; phase 1 (active, not recruiting)	
miR-155; Cobomarsen (MRG-106)	NCT03713320/phase 2 (recruiting)	T-cell lymphoma/mycosis fungoides
	NCT03837457/phase 2 (new/not yet recruiting)	

## Drug Resistance and miRNAs

A problem that continues to plague current treatment options in clinical medicine is drug resistance, including life-saving chemotherapeutics in patients with cancer. A number of published reports show that manipulating expression of specific miRNAs can alter the drug sensitivity or that miRNAs are themselves biologically involved in the body’s resistance response. Many of the standard therapies for breast cancer, such as Doxorubicin, Cisplatin, and Taxol, are all associated with deregulated miRNAs which may modulate resistance to the drug (Hu et al., 2018). Evidence suggests select miRNAs may even participate in treatments to breast cancer tumors that are specifically linked to high levels of estrogen receptors.

The role of miRNA transcripts in drug resistance is reportedly not restricted to cancer pathologies. The expression of miRNAs is associated with drug resistance in treatment of conditions such as epilepsy (Wang et al., 2015), multidrug-resistant (MDR) tuberculosis (Wagh et al., 2017), and insulin sensitivity (Gupta and Sandhir, 2018; Montastier et al., 2018). Notably, insulin sensitivity may not only impact the medical care of a diabetic patient, but studies also indicate its association with the onset of Alzheimer’s disease (AD) or other dementias. In fact, Mayo Clinic estimates approximately 80% of AD patients have Type 2 diabetes or insulin resistance (Kim and Feldman, 2015).

Specific mechanisms of drug resistance mediated by miRNAs are under ongoing investigation. Accumulating evidence, however, shows that the ATP-binding cassette (ABC) transporter family of proteins that activate drug resistance are regulated by miRNAs (Guo et al., 2018; Xie et al., 2018). The ABC family includes P-glycoprotein (Pgp), multidrug resistance-associated proteins (MRP), and breast cancer resistance proteins (BCRP), all of which can pump out drugs to create additional biological energy through ATP hydrolysis (Choi, 2005; Paskeviciute and Petrikaite, 2019). A recent report suggests miR-298 binds to the Pgp gene, thereby silencing the expression of Pgp and its ability to pump out antiepileptic drugs (Xie et al., 2018). In another recent study, restoration of miR-495 levels in non-small lung cancer cells reversed cisplatin resistance through a signaling pathway that represses levels of the ABCG2 protein (Guo et al., 2018). It is possible that co-administration of standard chemotherapeutics along with a select miRNA drug may help limit drug resistance in some cases through silencing of key proteins that directly promote low drug bioavailability or through alternate signaling pathways such as miR-155 in lung cancers (Van Roosbroeck et al., 2017; Bayraktar and Van Roosbroeck, 2018).

## Conclusion

As the pioneering discoveries from the Human Genome Project shift the landscape of clinical research, emerging miRNA therapeutics are offering new promise in health conditions that remain elusive to current treatment options. Development of bioinformatic programs for identification of miRNA-binding sites in target genes and their corresponding implicated biological pathways, along with an expanding platform of *in vitro* and *in vivo* preclinical research models, has helped expedite the translation of miRNAs into clinical medicine. The first siRNA human trial was conducted in 2004 and in 2018 the first siRNA drug was approved (Ozcan et al., 2015), paving the way for the miRNA class of transcripts that began to be actively investigated in basic biomedical research laboratories approximately 15 years ago. Indeed, as we now know the number of ncRNA transcripts is significantly greater than protein-coding genes, future human trials will undoubtedly expand their focus on epigenetic targets.

## Author Contributions

JK, JH, and GH wrote and edited the manuscript.

### Conflict of Interest Statement

GH is the CEO of Nova Clinical Research, LLC, and is not currently involved in any research projects involving miRNA.

The remaining authors declare that the research was conducted in the absence of any commercial or financial relationships that could be construed as a potential conflict of interest.

## References

[ref1] Bartel D. P. (2009). MicroRNAs: target recognition and regulatory functions. Cell 136, 215–233. 10.1016/j.cell.2009.01.002, PMID: 19167326 PMC3794896

[ref2] Bayraktar R. Van Roosbroeck K. (2018). miR-155 in cancer drug resistance and as target for miRNA-based therapeutics. Cancer Metastasis Rev. 37, 33–44. 10.1007/s10555-017-9724-7, PMID: 29282605

[ref3] Bayraktar R. Van Roosbroeck K. Calin G. A. (2017). Cell-to-cell communication: microRNAs as hormones. Mol. Oncol. 11, 1673–1686. 10.1002/1878-0261.12144, PMID: 29024380 PMC5709614

[ref4] Biswas S. (2018). MicroRNAs as therapeutic agents: the future of the battle against cancer. Curr. Top. Med. Chem. 18, 2544–2554. 10.2174/1568026619666181120121830, PMID: 30457051

[ref5] Buschmann D. Kirchner B. Hermann S. Marte M. Wurmser C. Brandes F.. (2018). Evaluation of serum extracellular vesicle isolation methods for profiling miRNAs by next-generation sequencing. J. Extracell. Vesicles 7:1481321. 10.1080/20013078.2018.1481321, PMID: 29887978 PMC5990937

[ref6] Chen Y. Gao D. Y. Huang L. (2015). In vivo delivery of miRNAs for cancer therapy: challenges and strategies. Adv. Drug Deliv. Rev. 81, 128–141. 10.1016/j.addr.2014.05.009, PMID: 24859533 PMC5009470

[ref7] Choi C. H. (2005). ABC transporters as multidrug resistance mechanisms and the development of chemosensitizers for their reversal. Cancer Cell Int. 5:30. 10.1186/1475-2867-5-30, PMID: 16202168 PMC1277830

[ref8] de Gooijer C. J. Baas P. Burgers J. A. (2018). Current chemotherapy strategies in malignant pleural mesothelioma. Transl. Lung Cancer Res. 7, 574–583. 10.21037/tlcr.2018.04.10, PMID: 30450296 PMC6204415

[ref9] Dong X. (2018). Current strategies for brain drug delivery. Theranostics 8, 1481–1493. 10.7150/thno.21254, PMID: 29556336 PMC5858162

[ref10] Elfimova N. Schlattjan M. Sowa J. P. Dienes H. P. Canbay A. Odenthal M. (2012). Circulating microRNAs: promising candidates serving as novel biomarkers of acute hepatitis. Front. Physiol. 3:476. 10.3389/fphys.2012.00476, PMID: 23267332 PMC3527896

[ref11] Gallo A. Tandon M. Alevizos I. Illei G. G. (2012). The majority of microRNAs detectable in serum and saliva is concentrated in exosomes. PLoS One 7:e30679. 10.1371/journal.pone.0030679, PMID: 22427800 PMC3302865

[ref12] Garg T. Bhandari S. Rath G. Goyal A. K. (2015). Current strategies for targeted delivery of bio-active drug molecules in the treatment of brain tumor. J. Drug Target. 23, 865–887. 10.3109/1061186X.2015.1029930, PMID: 25835469

[ref13] Ghosh D. Nandi S. Bhattacharjee S. (2018). Combination therapy to checkmate Glioblastoma: clinical challenges and advances. Clin. Transl. Med. 7:33. 10.1186/s40169-018-0211-8 30327965 PMC6191404

[ref14] Guo J. Jin D. Wu Y. Yang L. Du J. Gong K.. (2018). The miR 495-UBE2C-ABCG2/ERCC1 axis reverses cisplatin resistance by downregulating drug resistance genes in cisplatin-resistant non-small cell lung cancer cells. EBioMedicine 35, 204–221. 10.1016/j.ebiom.2018.08.001, PMID: 30146342 PMC6419862

[ref15] Gupta S. Sandhir R. (2018). Extending arms of insulin resistance from diabetes to Alzheimer’s disease: identification of potential therapeutic targets. CNS Neurol. Disord. Drug Targets 18, 172–184. 10.2174/1871527317666181114163515 30430949

[ref16] Hopkins A. L. (2008). Network pharmacology: the next paradigm in drug discovery. Nat. Chem. Biol. 4, 682–690. 10.1038/nchembio.118, PMID: 18936753

[ref17] Hu W. Tan C. He Y. Zhang G. Xu Y. Tang J. (2018). Functional miRNAs in breast cancer drug resistance. Onco. Targets Ther. 11, 1529–1541. 10.2147/OTT.S152462 29593419 PMC5865556

[ref18] International Human Genome Sequencing Consortium (2004). Finishing the euchromatic sequence of the human genome. Nature 431, 931–945. 10.1038/nature03001, PMID: 15496913

[ref19] Janssen H. L. Reesink H. W. Lawitz E. J. Zeuzem S. Rodriguez-Torres M. Patel K.. (2013). Treatment of HCV infection by targeting microRNA. N. Engl. J. Med. 368, 1685–1694. 10.1056/NEJMoa1209026, PMID: 23534542

[ref20] Kanlikilicer P. Rashed M. H. Bayraktar R. Mitra R. Ivan C. Aslan B.. (2016). Ubiquitous release of Exosomal tumor suppressor miR-6126 from ovarian cancer cells. Cancer Res. 76, 7194–7207. 10.1158/0008-5472.CAN-16-0714, PMID: 27742688 PMC5901763

[ref21] Karagiannis P. Takahashi K. Saito M. Yoshida Y. Okita K. Watanabe A.. (2019). Induced pluripotent stem cells and their use in human models of disease and development. Physiol. Rev. 99, 79–114. 10.1152/physrev.00039.2017, PMID: 30328784

[ref22] Kawaguchi T. Komatsu S. Ichikawa D. Tsujiura M. Takeshita H. Hirajima S.. (2016). Circulating MicroRNAs: a next-generation clinical biomarker for digestive system cancers. Int. J. Mol. Sci. 17. 10.3390/ijms17091459, PMID: 27598137 PMC5037738

[ref23] Kim B. Feldman E. L. (2015). Insulin resistance as a key link for the increased risk of cognitive impairment in the metabolic syndrome. Exp. Mol. Med. 47:e149. 10.1038/emm.2015.3, PMID: 25766618 PMC4351418

[ref24] Kocerha J. Kouri N. Baker M. Finch N. DeJesus-Hernandez M. Gonzalez J.. (2011). Altered microRNA expression in frontotemporal lobar degeneration with TDP-43 pathology caused by progranulin mutations. BMC Genomics 12:527. 10.1186/1471-2164-12-527, PMID: 22032330 PMC3229715

[ref25] Kola I. Landis J. (2004). Can the pharmaceutical industry reduce attrition rates? Nat. Rev. Drug Discov. 3, 711–715. 10.1038/nrd1470, PMID: 15286737

[ref26] Kristen A. V. Ajroud-Driss S. Conceicao I. Gorevic P. Kyriakides T. Obici L. (2018). Patisiran, an RNAi therapeutic for the treatment of hereditary transthyretin-mediated amyloidosis. Neurodegener Dis. Manag. 9, 5–23. 10.2217/nmt-2018-0033 30480471

[ref27] Lander E. S. Linton L. M. Birren B. Nusbaum C. Zody M. C. Baldwin J.. (2001). Initial sequencing and analysis of the human genome. Nature 409, 860–921. 10.1038/35057062, PMID: 11237011

[ref28] Lanford R. E. Hildebrandt-Eriksen E. S. Petri A. Persson R. Lindow M. Munk M. E.. (2010). Therapeutic silencing of microRNA-122 in primates with chronic hepatitis C virus infection. Science 327, 198–201. 10.1126/science.1178178, PMID: 19965718 PMC3436126

[ref29] Lekprasert P. Mayhew M. Ohler U. (2011). Assessing the utility of thermodynamic features for microRNA target prediction under relaxed seed and no conservation requirements. PLoS One 6:e20622. 10.1371/journal.pone.0020622, PMID: 21674004 PMC3108951

[ref30] Li Y. J. Xu M. Gao Z. H. Wang Y. Q. Yue Z. Zhang Y. X.. (2013). Alterations of serum levels of BDNF-related miRNAs in patients with depression. PLoS One 8:e63648. 10.1371/journal.pone.0063648, PMID: 23704927 PMC3660391

[ref31] McCall R. Miles M. Lascuna P. Burney B. Patel Z. Sidoran K. J.. (2017). Dual targeting of the cancer antioxidant network with 1,4-naphthoquinone fused gold(i) N-heterocyclic carbene complexes. Chem. Sci. 8, 5918–5929. 10.1039/c7sc02153d, PMID: 29619196 PMC5859730

[ref32] Mercatelli N. Coppola V. Bonci D. Miele F. Costantini A. Guadagnoli M.. (2008). The inhibition of the highly expressed miR-221 and miR-222 impairs the growth of prostate carcinoma xenografts in mice. PLoS One 3:e4029. 10.1371/journal.pone.0004029, PMID: 19107213 PMC2603596

[ref33] Montastier E. Beuzelin D. Martins F. Mir L. Marques M. A. Thalamas C. (2018). Niacin induces miR-502-3p expression which impairs insulin sensitivity in human adipocytes. Int. J. Obes. 10.1038/s41366-018-0260-5 30482933

[ref34] Ozcan G. Ozpolat B. Coleman R. L. Sood A. K. Lopez-Berestein G. (2015). Preclinical and clinical development of siRNA-based therapeutics. Adv. Drug Deliv. Rev. 87, 108–119. 10.1016/j.addr.2015.01.007, PMID: 25666164 PMC4504743

[ref35] Pammolli F. Magazzini L. Riccaboni M. (2011). The productivity crisis in pharmaceutical R&D. Nat. Rev. Drug Discov. 10, 428–438. 10.1038/nrd3405, PMID: 21629293

[ref36] Paskeviciute M. Petrikaite V. (2019). Overcoming transporter-mediated multidrug resistance in cancer: failures and achievements of the last decades. Drug Deliv. Transl. Res. 9, 379–393. 10.1007/s13346-018-0584-7, PMID: 30194528

[ref37] Rashed M. H. Kanlikilicer P. Rodriguez-Aguayo C. Pichler M. Bayraktar R. Bayraktar E.. (2017). Exosomal miR-940 maintains SRC-mediated oncogenic activity in cancer cells: a possible role for exosomal disposal of tumor suppressor miRNAs. Oncotarget 8, 20145–20164. 10.18632/oncotarget.15525, PMID: 28423620 PMC5386751

[ref38] Recchioni R. Marcheselli F. Olivieri F. Ricci S. Procopio A. D. Antonicelli R. (2013). Conventional and novel diagnostic biomarkers of acute myocardial infarction: a promising role for circulating microRNAs. Biomarkers 18, 547–558. 10.3109/1354750X.2013.833294, PMID: 24025051

[ref39] Reid G. Kao S. C. Pavlakis N. Brahmbhatt H. MacDiarmid J. Clarke S.. (2016). Clinical development of TargomiRs, a miRNA mimic-based treatment for patients with recurrent thoracic cancer. Epigenomics 8, 1079–1085. 10.2217/epi-2016-0035, PMID: 27185582

[ref40] Riffo-Campos A. L. Riquelme I. Brebi-Mieville P. (2016). Tools for sequence-based miRNA target prediction: what to choose? Int. J. Mol. Sci. 17. 10.3390/ijms17121987, PMID: 27941681 PMC5187787

[ref41] Scott K. A. Hoban A. E. Clarke G. Moloney G. M. Dinan T. G. Cryan J. F. (2015). Thinking small: towards microRNA-based therapeutics for anxiety disorders. Expert Opin. Investig. Drugs 24, 1–14. 10.1517/13543784.2014.997873 25566796

[ref42] St Laurent G. Wahlestedt C. Kapranov P. (2015). The landscape of long noncoding RNA classification. Trends Genet. 31, 239–251. 10.1016/j.tig.2015.03.007, PMID: 25869999 PMC4417002

[ref43] Sun X. Y. Lu J. Zhang L. Song H. T. Zhao L. Fan H. M. (2014). Aberrant microRNA expression in peripheral plasma and mononuclear cells as specific blood-based biomarkers in schizophrenia patients. J. Clin. Neurosci. 22, 570–574. 10.1016/j.jocn.2014.08.018 25487174

[ref44] Tsuji O. Sugai K. Yamaguchi R. Tashiro S. Nagoshi N. Kohyama J.. (2019). Concise review: laying the groundwork for a first-in-human study of an induced pluripotent stem cell-based intervention for spinal cord injury. Stem Cells 37, 6–13. 10.1002/stem.2926, PMID: 30371964 PMC7379555

[ref45] Van Roosbroeck K. Fanini F. Setoyama T. Ivan C. Rodriguez-Aguayo C. Fuentes-Mattei E.. (2017). Combining anti-Mir-155 with chemotherapy for the treatment of lung cancers. Clin. Cancer Res. 23, 2891–2904. 10.1158/1078-0432.CCR-16-1025, PMID: 27903673 PMC5449263

[ref46] van Zandwijk N. Pavlakis N. Kao S. C. Linton A. Boyer M. J. Clarke S.. (2017). Safety and activity of microRNA-loaded minicells in patients with recurrent malignant pleural mesothelioma: a first-in-man, phase 1, open-label, dose-escalation study. Lancet Oncol. 18, 1386–1396. 10.1016/S1470-2045(17)30621-6, PMID: 28870611

[ref47] Wagh V. Urhekar A. Modi D. (2017). Levels of microRNA miR-16 and miR-155 are altered in serum of patients with tuberculosis and associate with responses to therapy. Tuberculosis 102, 24–30. 10.1016/j.tube.2016.10.007, PMID: 28061948

[ref48] Wang R. Li N. Zhang Y. Ran Y. Pu J. (2011). Circulating microRNAs are promising novel biomarkers of acute myocardial infarction. Intern. Med. 50, 1789–1795. 10.2169/internalmedicine.50.5129, PMID: 21881276

[ref49] Wang J. Tan L. Tan L. Tian Y. Ma J. Tan C. C.. (2015). Circulating microRNAs are promising novel biomarkers for drug-resistant epilepsy. Sci. Rep. 5:10201. 10.1038/srep10201, PMID: 25984652 PMC4435024

[ref50] Washietl S. Will S. Hendrix D. A. Goff L. A. Rinn J. L. Berger B.. (2012). Computational analysis of noncoding RNAs. Wiley Interdiscip. Rev. RNA 3, 759–778. 10.1002/wrna.1134, PMID: 22991327 PMC3472101

[ref51] Weber J. A. Baxter D. H. Zhang S. Huang D. Y. Huang K. H. Lee M. J.. (2010). The microRNA spectrum in 12 body fluids. Clin. Chem. 56, 1733–1741. 10.1373/clinchem.2010.147405, PMID: 20847327 PMC4846276

[ref52] Weir D. W. Sturrock A. Leavitt B. R. (2011). Development of biomarkers for Huntington’s disease. Lancet Neurol. 10, 573–590. 10.1016/S1474-4422(11)70070-9, PMID: 21601164

[ref53] Xie Y. Shao Y. Deng X. Wang M. Chen Y. (2018). MicroRNA-298 reverses multidrug resistance to antiepileptic drugs by suppressing MDR1/P-gp expression in vitro. Front. Neurosci. 12:602. 10.3389/fnins.2018.00602, PMID: 30210283 PMC6121027

[ref54] Yang J. (2019). Patisiran for the treatment of hereditary transthyretin-mediated amyloidosis. Expert. Rev. Clin. Pharmacol. 12, 95–99. 10.1080/17512433.2019.1567326, PMID: 30644768

